# Measurements of methane and nitrous oxide in human breath and the development of UK scale emissions

**DOI:** 10.1371/journal.pone.0295157

**Published:** 2023-12-13

**Authors:** Ben Dawson, Julia Drewer, Toby Roberts, Peter Levy, Mathew Heal, Nicholas Cowan

**Affiliations:** 1 UK Centre for Ecology and Hydrology, Bush Estate, Midlothian, United Kingdom; 2 The University of Edinburgh, School of Chemistry, Edinburgh, United Kingdom; Tennessee State University, UNITED STATES

## Abstract

Exhaled human breath can contain small, elevated concentrations of methane (CH_4_) and nitrous oxide (N_2_O), both of which contribute to global warming. These emissions from humans are not well understood and are rarely quantified in global greenhouse gas inventories. This study investigated emissions of CH_4_ and N_2_O in human breath from 104 volunteers in the UK population, to better understand what drives these emissions and to quantify national-scale estimates. A total of 328 breath samples were collected, and age, sex, dietary preference, and smoking habits were recorded for every participant. The percentage of methane producers (MPs) identified in this study was 31%. The percentage of MPs was higher in older age groups with 25% of people under the age of 30 classified as MPs compared to 40% in the 30+ age group. Females (38%) were more likely to be MPs than males (25%), though overall concentrations emitted from both MP groups were similar. All participants were found to emit N_2_O in breath, though none of the factors investigated explained the differences in emissions. Dietary preference was not found to affect CH_4_ or N_2_O emissions from breath in this study. We estimate a total emission of 1.04 (0.86–1.40) Gg of CH_4_ and 0.069 (0.066–0.072) Gg of N_2_O in human breath annually in the UK, the equivalent of 53.9 (47.8–60.0) Gg of CO_2_. In terms of magnitude, these values are approximately 0.05% and 0.1% of the total emissions of CH_4_ and N_2_O reported in the UK national greenhouse gas inventories.

## Introduction

It has been reported that exhaled human breath can contain the greenhouse gases methane (CH_4_) and nitrous oxide (N_2_O) [1, 2], both of which have a much higher global warming potential than carbon dioxide (CO_2_) (34 and 265 for CH_4_ and N_2_O, respectively [3]). Where hydrocarbon chains (food types) are consumed by humans and turned into CH_4_ (and N_2_O from nitrogen intake), the global warming potential is no longer neutral, and human respiration has a net warming effect on the atmosphere. Due to their ruminant digestive system in which methanogenesis (biological production of CH_4_) occurs [4], herbivorous livestock (e.g., cattle) are known to emit large quantities of CH_4_ globally in the form of breath and flatus, accounting for approximately 20% of anthropogenic CH_4_ emissions [5, 6]. While the global biological mass of humans (390 Mt) is estimated to be similar in magnitude (~62%) to that of domesticated animals at the global scale [7], emissions of CH_4_ are assumed to be significantly smaller. However, few studies have been carried out to examine this explicitly, and no study to date has investigated emissions in breath of the UK population.

Methanogenic flora in the human gut produce CH_4_ that can be emitted via breath [1], flatus [8] and skin [9]. Humans are generally considered to be CH_4_ producers (MPs) if their breath concentration is more than 1 ppm above background concentration (~1.9 ppm [10]), and CH_4_ non-producers (MNPs) if not. However, there is evidence that all humans produce CH_4_ in breath to some extent [1] and those who do not exhale CH_4_ are still likely to release the gas in flatus [8]. Some studies have found that the propensity to produce CH_4_ in breath increases with age [11–13] and is higher among females [14–17], but there is contrary evidence for both these trends in the limited number of studies available. The highest reported proportion of MPs was found in African populations with up to 84% [15]. Proportions of MPs in Western populations vary from 25% [17] to 62% [18], while in Asian populations (such as Japanese) it can be as low as 15% [13]. The reasons for geographic, ethnic, diet, gender or age-based differences emissions of CH_4_ in human breath are not understood, and historical studies have likely suffered from issues such as poor detection limits of available analysers and limited sample populations (*n* < 100).

It is believed that N_2_O in human breath derives from the reduction of nitrates in food and water by denitrifying bacteria in the gut and oral cavity [2, 19, 20]. There is also evidence that endogenously produced nitric oxide (NO) is reduced to N_2_O by these bacteria [21]. Ingestion of nitrate-rich vegetables is reported to cause an increase in breath N_2_O concentration for up to 4 hours [19]; Petersen et al. (2015) [22] similarly highlighted that increasing nitrate in the diet of cattle can substantially increase N_2_O emissions in cattle breath. The number of studies on human N_2_O production is small, and thus there is no information on geographical or ethnic variation in breath concentrations, although an increase in concentration with age after childhood has been observed [20].

The predicted total emissions of these gases from humans is very small when compared to global emissions. Polag and Keppler (2019) [11] estimated that the global emission of CH_4_ from 7.5 billion people would be 0.41 ± 0.11 Tg CH_4_ yr^−1^. Mitsui (1997) [2] estimated that the global emission of N_2_O from 5.8 billion people would be up to 12 Gg N_2_O yr^-1^. This is the equivalent of approximately 0.11 and 0.16% of global anthropogenic emissions of CH_4_ and N_2_O, respectively (according to global estimates [6, 23]). Therefore, emissions of these gases are generally ignored in most environmental monitoring or inventory work as they are considered negligible. However, there are reasons to study these emissions further. The factors that affect human emissions of CH_4_ and N_2_O are not well understood and the impacts of an aging population and shifting diets is still relatively uncertain. Converting from high meat and protein content diets to higher fibre vegetarian options to mitigate emissions of greenhouse gases from meat production potentially results in higher production of gases in the human gut [24], and an element of pollution swapping could occur. In the UK, greenhouse gas measurements are carried out using top-down and bottom-up methods for validation purposes. Top-down measurements include the use of a “tall-tower” network where inverse modelling can be used to assess emissions of gases at a national scale [25]. The eddy covariance method is also used at tall urban tower sites to measure fluxes at large scales [26]. These measurement methods will observe emissions from a mixture of sources within their large footprints, and human breath is an unquantified factor that may add uncertainty to analysis, especially where human populations are dense. It is widely recognised that there is an offset between top-down and bottom-up emission inventories of greenhouse gases, believed to be as a result of missing or poorly quantified sources in bottom-up accounting methods [27]. Saunois et al. (2020) [6] report a difference greater than 20% between top-down and bottom-up estimates of global CH_4_ emissions.

The objectives of this study are (i) to quantify emissions of CH_4_ and N_2_O in human breath in the UK population, and (ii) to investigate factors that might affect the magnitude and variations in these concentrations. This study aims to identify patterns in emissions from individuals that may alter emission estimates in national scale accounting and provide a realistic national emission for the UK in particular.

## Method

### Study participation

A total of 328 breath samples were collected indoors in the city of Edinburgh from 104 volunteer participants between 12/12/2022 and 10/03/2023. All volunteers gave written consent to use the data and to publish the data in an anonymised format (Ethics approval was obtained from UKCEH Human Research Ethics Committee, HREC0009). Age, sex, dietary preference, and smoking habits were recorded for every participant (Table 1). The mean age of the participants across all samples collected was 30.2 and 35.8 years for males and females respectively. Information on the time of day, and whether participants had brushed their teeth, eaten, smoked, or exercised within 1 hour prior to measurement was available for 248 of the samples. Participants were asked if they had consumed meat, vegetables, fruit, salad, wheat, pulses, rice, egg, soya, oats, dairy, onion or garlic, potato and dried fruit. Details on the foods eaten within the 24 hours prior was collected for 170 samples. Forty-two participants gave more than one sample on different dates.

**Table 1:** A summary of male and female participants for age groups, dietary preferences, and smoking habits.

Factor	Number of Males	Number of Females	Total
Age 18–29 yr	41	23	64
Age 30–39 yr	8	11	19
Age 40–49 yr	4	7	11
Age 50–59 yr	5	2	7
Age 60+ yr	1	2	3
Meat-eaters	33	18	51
Vegetarians	10	7	17
Flexitarians*	16	20	36
Smokers	6	2	8
Undefined Diet	59	45	104

*Flexitarians identify themselves as being mostly vegetarian but will on occasion eat small quantities of meat.

### Breath analysis

For the collection of breath samples, 3 L Tedlar® gas-sampling bags were used. Participants were required to take in a deep breath and hold it for 5 s, then exhale approximately 80% of their lung capacity into the bag. Some participants needed to give a second breath to fill the sample bag. All bags were flushed with either 100% research grade nitrogen or with the participant’s breath prior to each sample collection. The double needle technique [28] was used to flush a 20 mL glass vial with 100 mL of breath sample extracted from the gas-sampling bags within 24 h of collection. These samples were analysed on an Agilent 7890B gas chromatograph (GC) with a flame ionisation detector (FID) and a micro electron capture detector (μECD) with nitrogen carrier gas, using an Agilent 7697A Headspace Autosampler for sample loading (Agilent, Santa Clara, CA, United States). Twenty-four atmospheric background measurements were taken across all locations at which breath samples were collected. Background concentrations of CO_2_, CH_4_ and N_2_O were defined as 1050, 2.0 and 0.34 ppm, respectively. Breath MPs were defined as 1 ppm above background concentration (3 ppm), as consistent with previous literature [29, 30].

### Statistical analysis

Data was analysed using the statistical software R, version 4.1.0 (R Core Team, 2021 [31]). Where data has a Gaussian distribution, uncertainties are reported as the 95% confidence interval around the mean. Where data are log-normally distributed, the method detailed by Zou et al. (2008) [32] is used to estimate the mean (Zou’s mean) with asymmetric 95% upper and lower confidence intervals, as implemented in the R package EnvStats [33]. Analysis was conducted using the exhaled breath concentrations minus the mean background concentration (negative values kept so as not to systematically bias data). All data referred to in the text is this concentration enhancement unless quoted as emitted concentration.

Estimated annual emissions from humans were calculated using an average breathing rate of 16 breaths per minute [34], an average lung tidal volume of 0.5 L [35], the ideal gas law under standard conditions, and the approximate current populations of 68.2 million for the UK and 8 billion for global estimates.

### Ethics statement

This study was reviewed by UKCEH’s Ethical Review Committee an approval was granted. Research was conducted in accordance with the principles embodied in the Declaration of Helsinki and in accordance with local statutory requirements. All participants have given written consent to collect and use the data collected in the study to publish this manuscript. All participants were over the age of 18 and consent from parents or guardians was not required.

## Results

**Fig 1:**
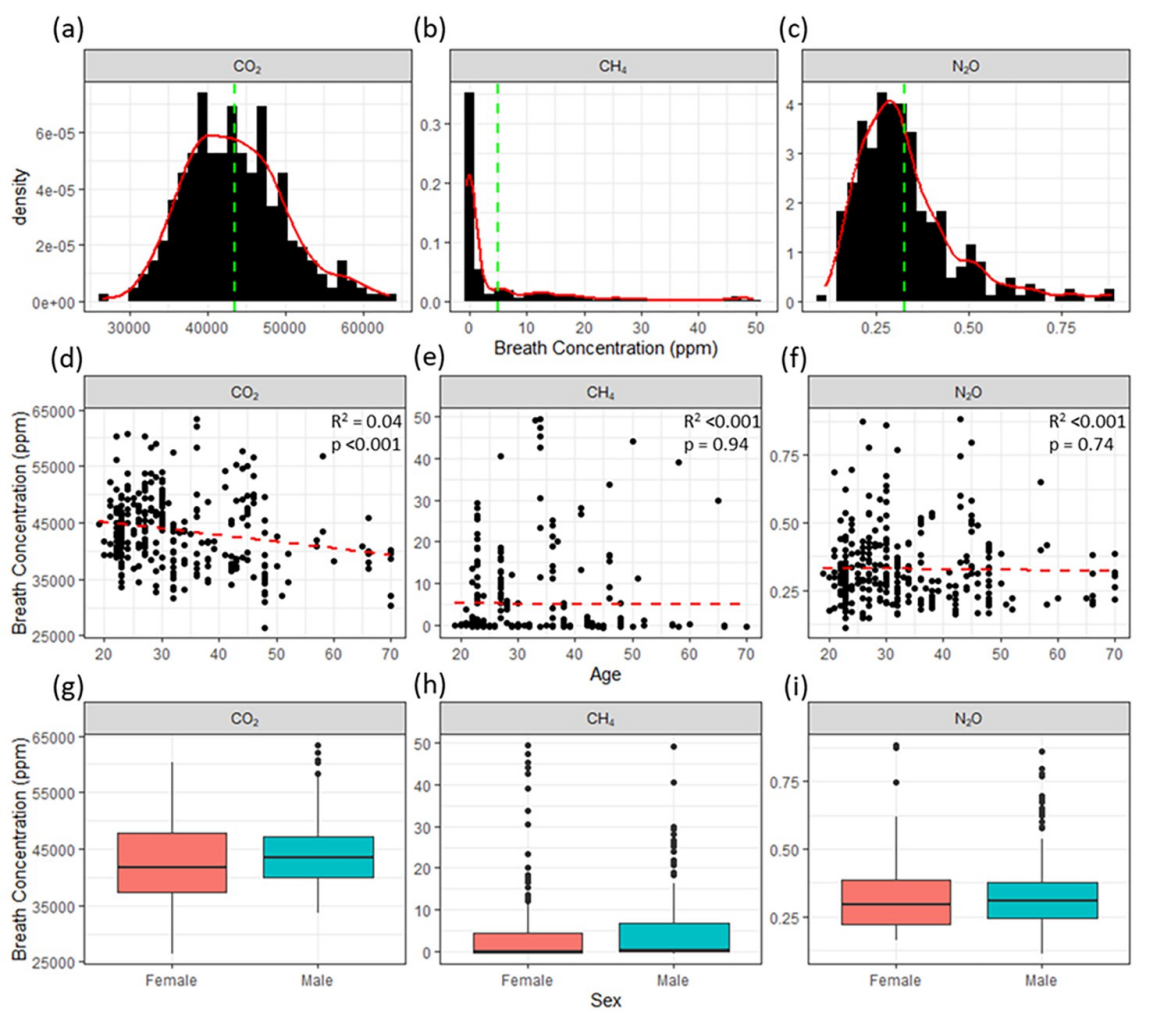
Density plots of (a) CO_2_, (b) CH_4_ and (c) N_2_O concentration enhancement in the breath of all participants, with mean concentrations shown as a green dashed line. (e, f) Concentration enhancement of gases in breath vs age, with linear fit and statistics (g-i) Concentration enhancement of gases in breath of female and male groups, presented as box & whisker plots with median and 25^th^ and 75^th^ percentiles.

Concentration enhancement of CO_2_ in the breath of the participants ranged from 26.5 to 63.4 parts per thousand (2.65–6.34%) following a Gaussian distribution, with an arithmetic mean of 4.35 (4.29–4.43) % (Fig 1A). All participants exhaled CO_2_, and while the data distribution skewed slightly towards higher values, overall, the data was relatively symmetrical around the mean. Concentration enhancement of CH_4_ in breath varied from -0.56 to 49.6 ppm, following a log-normal distribution with an arithmetic mean concentration of 5.08 and Zou’s mean of 4.26 (3.37–5.54) ppm (Fig 1B). While the lowest of these values is negative (emitted concentration below the 2 ppm background), the precision of the GC instrument is approximately 0.04 ppm (Drewer et al., 2021), thus the small number of negative values is likely to be instrumental noise as 55% of the concentration differences were less than the instrument precision. The distribution of concentration enhancement of CH_4_ are heavily skewed towards higher values, with a large number of concentrations near zero. A total of 32 (31%) of the participants were classed as MPs (single or mean concentrations in breath measurements exceeded the 3 ppm threshold). The arithmetic mean concentration enhancement of all samples measured from the MPs was 17.1 ppm and the Zou’s mean was 15.0 (11.9–19.9) ppm. The arithmetic emitted mean of the concentration enhancement among NMPs was -0.1 ppm, with most samples reporting breath concentrations around the precision limit of the GC instrument (results from NMPs were essentially instrumental noise around zero). Therefore, it is approximated that 31% of people emitted a mean concentration of 15.0 (11.9–19.9) ppm CH_4_ in their breath, while the rest emit effectively none. Concentration enhancement of N_2_O in the breath of the participants also followed a log-normal distribution, ranging from 0.11 to 0.88 ppm with an arithmetic mean of 0.33 and a Zou’s mean of 0.329 (0.315–0.342) ppm (Fig 1C). The skew in the distribution of N_2_O concentrations was more towards higher values than the CO_2_ distribution, but not as extreme as that of CH_4_ concentrations. No concentration enhancements of N_2_O in breath were below background levels, indicating that while there is a large variation in observed N_2_O concentrations, all participants emitted at least some N_2_O.

### Impact of demographics

The majority of the participants (62%) in the study were below the age of 30 (*n* = 64), though each decadal age grouping contained several participants (Fig 1D–1F). No strong relationships were observed between age and emission of CH_4_ and N_2_O in breath between age and gas concentrations, though due to a lack of participants in the later age groups, we cannot prove this definitively with this data set. Due to the potential bias of having a larger number of younger participants and the impact of the log-normal distribution of data, we split the data into groupings to clarify comparisons in age. When the participants were split into two groupings, either side of a 30-year-old threshold (arbitrary split to balance age groups with n = 64:40), a difference in CH_4_ emissions was observed between the groups. The relative ratio of MPs in the 30yr+ group (total *n* = 40) was 40%, larger than the number of the 25% observed in the <30yr group. The Zou’s mean concentrations observed in the <30yr and 30yr+ age groups was 4.3 (3.1–5.5) ppm and 10.4 (5.9–14.8) ppm, respectively, and Zou’s mean concentrations in the breath of the MPs only in these groupings was 10.7 (8.7–13.8) ppm and 19.14 (13.5–30.6) ppm, respectively. Emissions of N_2_O showed no correlation with age (p = 0.74).

There were no clear differences between mean emissions from male and females (Fig 1G–1I). The proportion of female and male participants classed as MPs was 38% and 25%, respectively. However, there was no obvious difference between the measured mean concentrations for each sex overall or among MPs. Only 9 of the participants were smokers which prevented meaningful statistical analysis with the other participants; however, no notable differences in emissions of the three GHGs investigated were observed in these samples.

### Impact of diet

**Fig 2:**
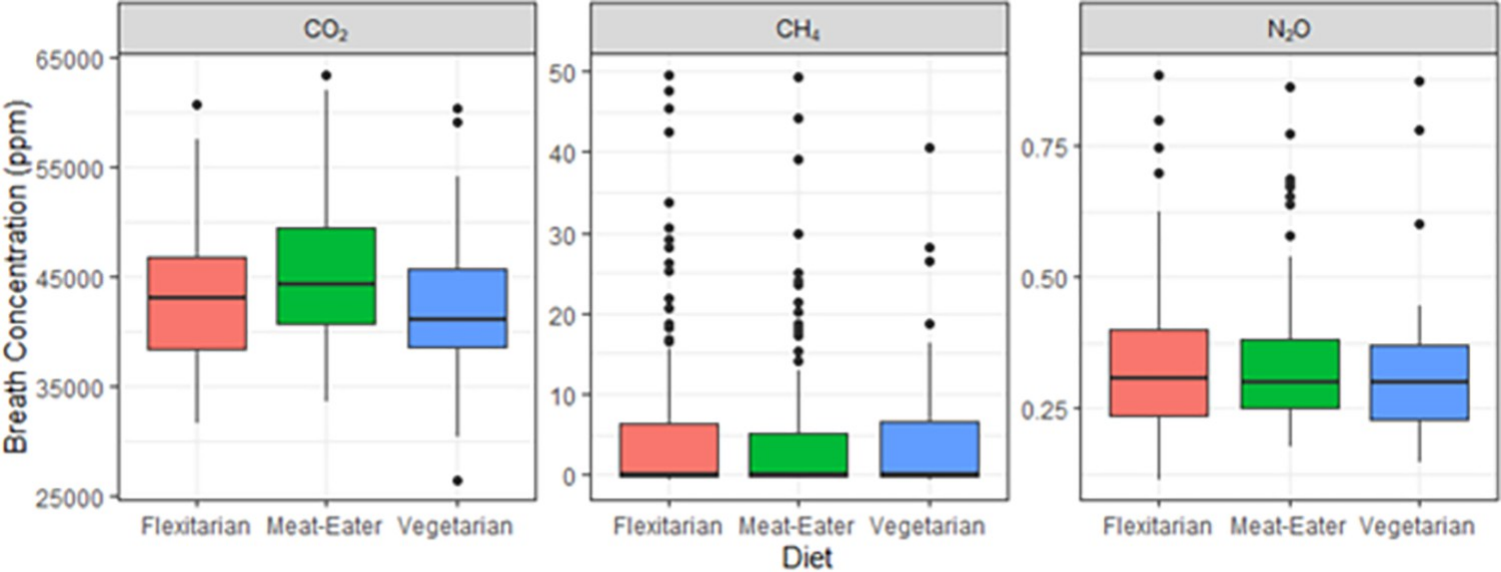
Concentrations of gases in breath (above background) of different dietary groupings, presented as box plots, with median and 25th and 75th percentiles.

**Fig 3:**
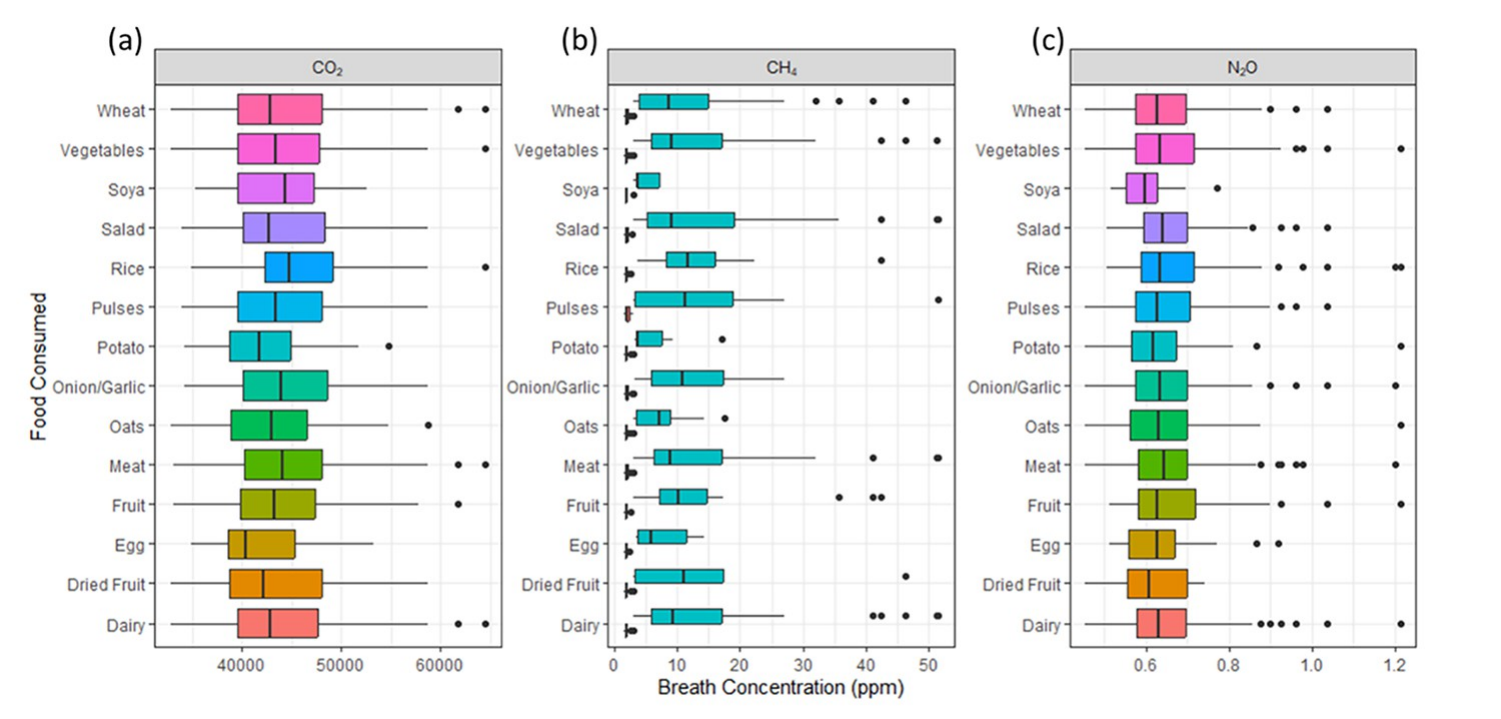
Concentrations of gases (above background) in the breath of participants who had consumed the listed food type in the 24 h prior to sampling. Data is presented as box plots, with median and 25th and 75th percentiles. In (b), the emissions of methane are split into MP and NMP categories.

Samples from participants in this study were separated into three dietary groupings: those who ate meat regularly (meat eater, sample *n* = 119), those who eat meat up to twice a week (flexitarian, sample *n* = 145) and those who ate no meat at all (vegetarian, sample *n* = 64). No trends were observed between the emissions of all 3 greenhouse gases with any of the three dietary groupings in this study (Fig 2). Further investigation into foods consumed 24 h prior to breath sampling also provided no trends with observed emissions (Fig 3). Many of these groups overlapped due to dietary variation over a 24 h period. When split into MP and NMP populations (Fig 3A), there is still no strong correlation between diet and observed CH_4_ emissions. A reduction of approximately 20% in N_2_O emissions was observed in the breath of participants who had provided breath samples prior-to and shortly after brushing teeth; however, reductions were inconsistent and dependent upon the magnitude of the emissions from the participant.

## Discussion

### Assessment of sampling method

At rest, the normal CO_2_ concentration in human breath is around 4.0% [36], which is slightly lower than the concentration observed in this study of 4.4 (4.3–4.5) %. However, the breath collection method in this project was designed for measuring CH_4_ and N_2_O concentrations consistently, not for CO_2_ respiration rates. The likely cause for the skew in CO_2_ concentrations on the higher end of the scale is that some participants took slightly longer to blow into the bag than others, increasing the CO_2_ in breath which was held longer as a result. To test if the skew in N_2_O and CH_4_ concentration enhancement was due to the sampling method, a CO_2_ correction was applied as a check. Here, the CH_4_ and N_2_O concentrations were multiplied by the ratio of the mean measured CO_2_ concentration divided by the CO_2_ measured in individual samples. The skew in CH_4_ and N_2_O concentrations remained relatively unchanged by the correction, hence the concentrations were not correlated with longer breath holding and the skew in both data sets is real. There was also no correlation between the concentration enhancement of any of the gases with each other, suggesting that samples with highly skewed concentrations were independent of the method and representative of real differences between individuals.

### Impact of demographics

Emissions of CH_4_ in breath from a given human population depends largely on the number of MPs present in the demographic. The percentage of MPs identified in this study (31%) is at the lower end of literature values for western populations (25–62% [8, 17, 18, 37]). One reason for this may be the lack of participants in older age groups in this study. The 25% of MPs in the <30 yr age group is consistent with percentages reported for similar age groups in the west [14, 38]. The higher percentage of MPs in the older age group is also consistent with the literature, with most previous studies finding an overall increase in the percentage of MPs with age [14, 38]. While previous studies have identified higher ratios of MPs in older age groups, most prior studies have either not reported concentration trends within MPs with age or have found no trend [12]. The finding of higher breath concentration of CH_4_ among MPs in the 30 yr+ grouping in this study has not been previously observed.

The results reported in this study are consistent with most previous studies that found a higher percentage of MPs in females (38%) when compared to males (25%) [12, 14, 16, 17]. It is also consistent in not finding any difference between the mean concentrations among MPs of both genders. It appears that females are more likely to be MPs, but those who are MPs do not exhale more CH_4_ than male MPs. We are unable to offer a reason for the difference in proportion of MPs between genders in this and other studies.

It has been reported in previous studies that region of birth or ethnicity is a strong indicator of the likelihood to be an MP, with African populations [15] much more likely to be MPs than Asian populations [13]. It was a limitation of this study that information on ethnicity or place of birth was not collected, but this data is the only reported from a population within the UK since McKay et al. (1985) [8]. There is evidence that MP status is determined in early childhood [16], and that the mother’s MP status is a strong indicator [38], but that the familial link is not genetic [16]. MP status may be determined in early childhood through acquiring the methanogenic bacteria via diet or breast milk, but it appears that it takes time for the bacteria to reach a critical population and thus for the MP status to develop, which is the reason for an increase in the percentage of MPs with age [39]. Our finding of increased breath concentration in older MPs also fits this theory, because if methanogenic bacteria become more established in the digestive system throughout a lifetime, then breath CH_4_ concentration would also increase. It may be that this is limited by a number of other factors, such as food consumption, individual health and other impacts that affect breath CH_4_ concentration enhancement.

The mean breath N_2_O emission concentration enhancement of 0.33 ppm is consistent with some previous studies (Mitsui et al., 1997, Mitsui and Kondo, 1998). Some studies classified people as breath N_2_O producers and non-producers in the same way as is done for CH_4_, with a cut-off of 0.1 ppm above background concentration (Mitsui et al., 1997, Mitsui and Kondo, 1999). However, in this study none of the samples given were below this cut-off and thus every person would be an N_2_O producer. Given the evidence that humans endogenously produce NO (Palmer et al., 1987), it is plausible that all humans emit N_2_O through reduction of NO by denitrifying bacteria in their gut and oral cavity, but the concentration enhancement was too small to be detected by previous instruments.

There have been no previous reported differences in breath N_2_O concentration between sexes in previous studies, which is consistent with our findings. Higher concentration enhancements have been found in older people in Japan (Mitsui et al., 1997, Mitsui and Kondo, 1998, Mitsui and Kondo, 1999); however, the results of this study show no such trend.

### Impact of diet

This study attempted to identify foods that affect breath CH_4_ and N_2_O concentration enhancement without interfering with typical dietary behaviour; however, no trends were identified. Breath CH_4_ concentration has been reported to increase on ingestion of lactulose [18] and have a positive correlation with total dietary fibre [38]. This study found no increase in breath CH_4_ concentration of those who had eaten dairy and did not collect information on dietary fibre intake. Mitsui and Kondo (1999) [19] reported increased breath N_2_O concentrations for 4 h after ingestion of nitrate-rich vegetables. In this study, difference in concentration of N_2_O related to any of the foods tested was found. Due to the magnitude of the random variance in emissions measured form the participants in this study, it is highly likely that a full investigation into whether particular diets have an impact on CH_4_ and N_2_O emissions requires a dedicated experiment on each food type with a large number of participants and strict diet regimes. Another limitation of the study design in this case is that measurements were taken during winter months only, and diet or other unforeseen seasonal environmental factors may alter human breath emissions to some extent. The purpose of this study was exploratory, to determine if certain generic diets had an overall impact on an individual’s emissions of these gases, which does not seem to be the case. Concentration enhancement of both CH_4_ and N_2_O in the breath of vegetarians and meat consumers are similar in magnitude. Based on these results, we can state that, when estimating emissions from a population within the UK, diet or future diet changes are unlikely to be important when estimating emissions across the UK as a whole.

### UK and global-scale emissions

The results in this study suggest that when considering CH_4_ production in human breath in larger populations, only age and gender are relevant factors in determining the quantity of MPs, and thus the total CH_4_ emissions. The number of people below 30 years of age accounts for 35.5% of the UK population, which is currently 68.2 million. An estimated 51% of people below the age of 30 are male, and 49% of people above the age of 30 are male [40]. As no demographic or dietary factors were found to correlate with N_2_O emissions in breath, a single population factor was used to calculate N_2_O emissions. Based on estimates of approximately 4205 m^3^ of breath exhaled per person and using the ratio of MPs in each grouping in this study multiplied by demographics of the UK, we estimate emissions for the UK (Table 2). We estimate a total emission of 1.04 (0.86–1.40) Gg of CH_4_ and 0.069 (0.066–0.072) kt of N_2_O in human breath annually in the UK, the equivalent of 59.39 Gg of CO_2_. In terms of magnitude, these values are approximately 0.05% and 0.1% of the total emissions of CH_4_ and N_2_O reported in the UK national greenhouse gas inventories [41].

**Table 2:** Estimates of CH_4_ and N_2_O emissions from breath of all inhabitants of the UK. Demographic data sourced from ONS, 2023. Global warming potential of 34 and 265 used to estimate CO_2eq_ for CH_4_ and N_2_O, respectively; Sixth Assessment Report, IPCC 2022 [3].

Gas	Age	Gender	Pop (x10^6^)	MP (%)	MP Pop (x10^6^)	Breath Conc. (ppm)	Emission (Gg)	CO_2_ equivalent (Gg)
CH_4_	<30 yr	Female	11.8	39	4.6	10.7 (8.7–13.8)	0.14 (0.12–0.19)	5.0 (4.0–6.4)
	30+ yr	Female	22.6	41	9.2	19.14 (13.5–30.6)	0.52 (0.37–0.84)	17.9 (12.6–28.7)
	<30 yr	Male	12.3	24	3.0	10.7 (8.7–13.8)	0.09 (0.07–0.12)	3.2 (2.6–4.2)
	30+ yr	Male	21.4	22	4.7	19.14 (13.5–30.6)	0.26 (0.19–0.43)	9.1 (6.4–14.6)
						**Total CH**_**4**_	1.04 (0.86–1.40)	35.4 (29.3–47.6)
N_2_O	All	All	68.2			0.329 (0.315–0.342)	0.069 (0.066–0.072)	18.4 (17.7–19.2)
							**Total All**	53.9 (47.8–60.0)

Based on the mean concentrations of 15.0 (11.9–19.9) ppm in the breath of MPs, which accounted for 31% of participants in this study, a global total emission of 0.11 (0.09–0.15) Tg yr^-1^ of CH_4_ is very approximately estimated for breath emissions at a global scale (assuming a population of 8 billion). This is considerably lower than some previous estimates (e.g. 0.4 Tg yr^-1^ reported by Polag and Keppler, 2019) [11], which may be due to the relatively low number of MPs in the participant group in this study. The variation geographically and demographically of the ratio of MPs is still not understood, and there remain large areas of the world with no data. Polag and Keppler (2019) [11] predict 1.2 Tg yr^-1^ of CH_4_ in human emission by the year 2100 using a weighted estimation on age, sex, and geographical variance in population and MP percentage. If the finding here of an increase in breath concentration with age among MPs is also true, this value may be higher.

With a population of 8.9 million people, it could be assumed that CH_4_ emissions in human breath in the greater London area is approximately 0.14 Tg CH_4_ yr^-1^. With an area coverage of 1569 km^2^ we estimate an average annual flux of 0.09 tons CH_4_ km^-2^ yr^-1^, which is negligible compared to the 72 tons CH_4_ km^-2^ yr^-1^ reported by Helfter et al. (2016) [26] for the region. While there is an extremely high concentration of people in cities compared with more rural areas, the emissions of CH_4_ associated with fossil-fuel burning, gas leaks and wastewater leakage in cities are several orders of magnitude greater than that from breath. Natural soils can be a source or sink of CH_4_ in the UK, with typical grasslands emitting approximately 0.1 tons CH_4_ km^-2^ yr^-1^ (0.19 nmol m^−2^ s^−1^) on average [42]. For comparative purposes only, if the Greater London area were a managed grassland, the soil emissions of CH_4_ would be equivalent to that of human breath in the same area.

The estimated annual global emissions of N_2_O of approximately 0.01 Tg yr^-1^ is similar in value to the 0.012 Tg yr^-1^ estimated by Mitsui et al. (1997) [2]. While total emissions of N_2_O in breath are relatively negligible, the drivers behind the large spread in observed concentrations remains poorly studied and unexplained. Human flatus has been found with CH_4_ content of up to 29% [43], but very little is known about how this varies. Based on some opportunistic lab work that was carried out in this study, we know that flatus can contain extremely high concentration of N_2_O (greater than 30 ppm), but this has never been properly studied or reported in literature for humans. While flatus from livestock is commonly measured, there are barriers when performing experimentation on humans, primarily the embarrassment of participants and the inability to secure funds to carry out such research which carries with it some stigma due to the nature of the task. As the estimates of CH_4_ and N_2_O presented in this study do not account for flatus, we represent only the lowest possible emission from humans, and the true value of our own bodily emissions are likely significantly higher as a species.

Petersen et al. (2015) [22] reported that 2.7 mg hr^-1^ of N_2_O was emitted per head of cattle based on experimental evidence. Based on livestock unit (LSU) conversions of 0.1 for sheep and 0.8 for pigs, we can attribute a hypothetical N_2_O emission rate of 0.27 and 2.16 mg hr^-1^ for sheep and 0.8 for pigs, respectively. In the UK, a total of 9.6 million cattle, 22 million sheep and 5 million pigs are recorded [44], which using the previous estimates would generate approximately 0.37 Gg of N_2_O per year. Based on pet number estimates of 11 million dogs and 11 million cats [45] we can estimate more N_2_O in breath and flatus, though this has never been measured. Further wild mammal population such as deer, badgers, foxes and rodents would also add to this total, though these values are also unknown. While each of these individual sources is small, combined it is possible that emissions add up, and could exceed 1% of total N_2_O emissions in the UK (approximately 0.7 Gg N_2_O). Emissions of N_2_O in breath and flatus of mammals is not included in any greenhouse gas inventory or model as it is assumed to be negligible, but this may not be the case. The addition of nitrates to animal diets has been shown to reduce CH_4_ emissions, but these dietary changes could drastically increase N_2_O in the breath of livestock [22]. We recommend further exploratory work to quantify and understand N_2_O emissions from breath and flatus in the livestock sector, especially in regard to feeding nitrates to animals to reduce CH_4_ emissions, which is considered one option to reducing the carbon footprint of cattle in particular [46, 47].

## Conclusions

The measurements carried out in this study allow us to estimate UK-scale emissions of approximately 1.04 (0.86–1.40) Gg of CH_4_ and 0.069 (0.066–0.072) Gg of N_2_O emitted in the form of human breath. Based on a sample population of 104 volunteers, we estimate that the methane producing (MP) population in the UK is 25% for those aged less than 30 years, and 40% for those aged over 30 years of age. We have found no correlation between diet and emission of CH_4_ and N_2_O in breath and recommend if future studies wish to assess this in more detail, that rigid dietary regimes are implemented to reduce the effect of heterogeneity of emissions in a given population. While emissions of CH_4_ and N_2_O account for only 0.05% and 0.1% of the total emissions in the UK national greenhouse gas inventories, respectively, we would urge caution in the assumption that emissions from humans are negligible. We report only emissions in breath in this study, and flatus emissions are likely to increase these values significantly, though no literature characterises these emissions for people in the UK. Assuming that livestock and other wild animals also exhale emissions of N_2_O, there may still be a small but significant unaccounted for source of N_2_O emissions in the UK, which could account for more than 1% of national-scale emissions.

## Supporting information

S1 Data (DOCX)

S2 Data (CSV)
