# Supplementary material for: Measurements of methane and nitrous oxide in human breath and the development of UK scale emissions
Source: PLoS One. 2023 Dec 13;18(12):e0295157. doi: 10.1371/journal.pone.0295157 (PMC10718453; doi:10.1371/journal.pone.0295157)
Supplement: S1 Data — (DOCX) [file pone.0295157.s001.docx]

**Measurements of methane and nitrous oxide in human breath and the development of UK scale emissions**

**Supplementary Materials:** Breath gas concentration dataset

Ben Dawson, Julia Drewer, Toby Roberts, Peter E. Levy, Mathew R. Heal, Nicholas Cowan

**Corresponding Author:** Nicholas Cowan (nicwan11@ceh.ac.uk)

A total of 328 breath samples were collected indoors in the city of Edinburgh from 104 volunteer participants between 12/12/2022 and 10/03/2023. All volunteers gave written consent to use the data and to publish the data in an anonymised format (Ethics approval was obtained from UKCEH Human Research Ethics Committee, HREC0009).

NA values in the dataset represent where ancillary information was not collected with a sample.

| **Column Header** | **Units** | **Description** |
| --- | --- | --- |
| Compound.Name | NA | ID of GHG gas measured |
| Gas.Conc | ppm | Concentration of GHG in breath sample |
| Date.of.Birth | Date | Date of birth of the person providing the sample |
| Gender | NA | Gender (sex) of the person providing the sample |
| Smoker | NA | Is the subject a smoker |
| Typical.Daily.Meat.Intake | g | Estimated daily meat consumption |
| Dietary.Preference | NA | Dietary preference of the person providing the sample |
| Date | Date | Date sample was collected |
| Time | Time of Day | Time of day sample was collected |
| Meat, Vegetables, Fruit, Salad, Wheat, Pulses, Rice, Egg, Soya Oats, Dairy, Onion.and.Garlic, Potato, Dried.Fruit | True/False | Had any of these food types been consumed within 24 h of the sample being given (1 = True) |
